# Comprehensive Assessment of Risk Factors of Cause-Specific Infant Deaths in Japan

**DOI:** 10.2188/jea.JE20160188

**Published:** 2018-06-05

**Authors:** Yui Yamaoka, Naho Morisaki, Haruko Noguchi, Hideto Takahashi, Nanako Tamiya

**Affiliations:** 1 Department of Health Services Research, Faculty of Medicine, University of Tsukuba, Ibaraki, Japan; 2 Department of Social Medicine, National Center for Child Health and Development, Tokyo, Japan; 3 Faculty of Political Science and Economics, Waseda University, Tokyo, Japan; 4 Office of Information Management and Statistics, Radiation Medical Science Center for the Fukushima Health Management Survey, Faculty of Medicine, Fukushima Medical University, Fukushima, Japan

**Keywords:** infant death, vital statistics, risk factor, unintentional injury, intentional injury

## Abstract

**Background:**

Public attention is given to infants with socially high risks of child abuse and neglect, while clinical attention is provided to infants with a biologically high risk of diseases. However, few studies have systematically evaluated how biological or social factors cross over and affect cause-specific infant mortality.

**Methods:**

We linked birth data with death data from the Japanese national vital statistics database for all infants born from 2003–2010. Using multivariate logistic regression, we examined the association between biological and social factors and infant mortality due to medical causes (internal causes), abuse (intentional external causes), and accidents (unintentional external causes).

**Results:**

Of 8,941,501 births, 23,400 (0.26%) infants died by 1 year of age, with 21,884 (93.5%) due to internal causes, 175 (0.75%) due to intentional external causes, and 1,194 (5.1%) due to unintentional external causes. Infants with high social risk (teenage mothers, non-Japanese mothers, single mothers, unemployed household, four or more children in the household, or birth outside of health care facility) had higher risk of death by intentional, unintentional, and internal causes. Infant born with small for gestational age and preterm had higher risks of deaths by internal and unintentional causes, but not by intentional causes.

**Conclusions:**

Both biological as well as social factors were associated with infant deaths due to internal and external causes. Interdisciplinary support from both public health and clinical-care professionals is needed for infants with high social or biological risk to prevent disease and injury.

## INTRODUCTION

Infant death, in many cases, is not due to one single cause but to an unfortunate complex assembly of risk factors.^1^ Many studies on infant mortality have focused on either social factors and their relation to child abuse and neglect,^2^^–^^4^ or on biological risk factors, such as low birth weight, preterm birth, congenital abnormalities, or other perinatal medical complications, and their association with death due to medical causes.^5^^,^^6^

However, the effects of biological and social risk factors on infant death may intercorrelate with each other. Prematurity of infants increases the likelihood of child protection service registration.^7^ Parents of infants with biological risks tend to face difficulties in parenting because of psychological distress^8^^,^^9^ or postpartum depression.^10^ Families with social risk may have difficulty in parenting, so their infants may have a higher risk of becoming severely sick.^11^^–^^15^

Comprehensive assessment of biological and social risk factors of cause-specific infant death will be useful to detect and provide adequate support to infants who have multiple risk factors, thereby reducing risk of subsequent deaths. However, no study has comprehensively and simultaneously assessed how biological and social risk factors relate to deaths due to medical causes, accidents, and abuse. Thus, we aimed to assess how biological and social risk factors were related to cause-specific infant death using a population-based database in Japan.

## METHODS

### Data source and linkage process

The Japanese vital statistics database was established in 1899 in accordance with the Family Registry Law and Provisions Regarding Notification of Stillbirths and is maintained by the Ministry of Health, Labour and Welfare. We used birth and infant death data recorded in the database for the period 2003–2011. This starting year was selected because the 10th revision of the International Classification of Diseases (ICD-10) was introduced in 2003 and updated the ICD-9.

Birth and death records are not linked in the database and do not have unique identifiers. Due to limited data quality on birth characteristics included in the death records, deterministic linkage using common variables between the two datasets was only able to link 88% of the death certificates to a birth certificate. Thus, we used the probabilistic linkage method developed by Fellegi and Sunter^16^ to link the two sets of data. This linkage method uses multiple common variables (in our case, date of birth, mother’s date of birth, gestational week of delivery, birth weight, multiplicity, birth order, sex, and nationality) to calculate the likelihood that two records are a true match, and assigns matches based on this probability. Such data linkage algorithms have been shown to be powerful and have been implemented in studies in the United States^17^ and Australia.^18^ Detailed methodology is described in Appendix 1.

We matched 25,413 out of 25,451 (99.96%) infant death certificates to their corresponding birth certificates, of which there were 100,175,174. As we did not have data on infant deaths in 2012, which would include infant deaths among those born in 2011, we excluded all births in 2011 and limited our analysis to 8,941,501 births in the period of 2003–2010.

### Classification of infant death

Our main outcome of interest was cause-specific infant death. We utilized ICD-10 codes (V01–Y98), which indicated external causes of mortality. External causes were sub-classified into unintentional injury (V01–X59, Y85–86), intentional injury (X85–Y09, Y87.1), and undetermined injury (injury for which we could not judge the presence of intention) (Y10–Y34, Y87.2, Y89.9) in line with previous studies.^19^^,^^20^

### Definition of variables

We categorized maternal and paternal age into ≤19, 20–24, 25–29, 30–34, 35–39, and ≥40 years; place of residence as a government-designated city (population over 500,000), other city (population 30,000–500,000), or a town or village (population under 30,000), based on the official classification by name of municipal governments; and family employment status at birth as follows: employed, self-employed, agricultural work or irregular employed, and unemployed. The number of children in the household was categorized into 1, 2–3, and ≥4 children. Single mothers included unmarried, divorced, and widowed mothers. We defined preterm birth as birth before 37 completed weeks of gestation. Small for gestational age (SGA) was defined as a birth weight lower than the 10th percentile of the Japanese gestational age-specific birth weight standards.^21^

We defined the following as biological risk factors of infant death: male infant, multiple births, SGA, preterm delivery, previous experience of stillbirth, and older maternal age (40 and older). Similarly, we defined the following as social risk factors of infant death: younger maternal age (19 and younger), non-Japanese mother, birth outside of a health care facility, single mother, unemployed household, and four or more children in the household.

### Statistical analysis

First we compared infant, parent, and household characteristics between infants who were alive at age 1 year and those who had died from internal or external causes using chi-square test. Second, we compared cause-specific mortality by infant age in days or weeks. We used Cuzick’s non-parametric trend test to examine the association between increasing age (every 4 weeks) and mortality, as well as with mortality due to internal and external causes. For neonatal deaths, we also observed whether differences existed between timing of death (day 0, day 1, days 2–6, and days 7–27) and cause of death using the chi-square’s test, or Fisher’s exact test if the numbers were small and included an expected cell of less than five.

Third, we conducted multivariable nominal logistic regression analysis to examine independent associations of risk factors related to death by both internal and external causes, the latter of which was also subdivided into unintentional injury, intentional injury, and undetermined injury. This analysis was conducted separately for single and multiple births, as multiple births are more likely to be due to fertility treatments, which are related to parental social backgrounds, and multiplicity is a well-known risk factor for biological problems of the infant, including prematurity and SGA.^22^^,^^23^ All multivariable models included infant, mother, and household characteristics as examined in the univariate analysis except parity, which was highly correlated with the number of children in the household. Place of residence and year of birth were also included in the models to account for regional differences and secular changes in healthcare.

Lastly, we focused on maternal age, a well-known social factor associated with unintentional or intentional injury in many studies,^24^^,^^25^ and analyzed its associations with infant mortality at different life stages (day 0–1, day 2 to <4 weeks, 4 weeks to <12 weeks, 12 weeks to <24 weeks, and 24–52 weeks) using multivariable logistic regression.

For data linkage we used LinkPlus (Center for Disease Control and Prevention, Atlanta, GA, USA), and for all other analyses, we used STATA/MP, version 14.0 (Stata Corp LP, College Station, TX, USA).

### Ethical considerations

This study was approved by the official ethics review board of the University of Tsukuba (Document No. 1009, 10/01/2015). Authors obtained permission for secondary use of information from the vital statistics according to Article 33 of the Statistics Act, which states that researchers may utilize questionnaire information pertaining to statistical surveys provided that the study protocol is based on a governmental grant and the findings would contribute to the development of academic research.

## RESULTS

Overall, of the 8,941,501 infants born in the period from 2003 through 2010, 23,400 (0.26%) died before their first birthday, with an average infant mortality rate (IMR) of 2.62 per 1,000 live births. Internal causes accounted for 93.5% of total infant deaths (*n* = 21,884, 2.45/1,000 live births); unintentional injury covered 78.8% of external causes (*n* = 1,194, 0.13/1,000 live births); intentional injury (*n* = 175) accounted for 11.5% of external causes and 0.75% of all infant deaths, and a small number of external deaths related to medical treatments or medications (*n* = 29) accounted for the remaining deaths due to external causes (Table 1).

**Table 1.** Cause of death among 8,941,501 infants born from 2003–2010

	*n*	/1,000 live births
All deaths	23,400	2.62
Death by internal causes	21,884	2.45
Death by external causes	1,516	0.17
Unintentional injury	1,194	0.13
Intentional injury	175	0.020
Undetermined injury	118	0.013
Medical-related death	29	0.0032

In Table 2 we show infant, parental, and household characteristics by cause of death. Among parental and household characteristics, non-Japanese mothers, single mothers, unemployed household, four or more children in the household, and previous history of stillbirth were significantly associated with death due to both internal and external causes. Having a father of non-Japanese nationality was significantly associated with death due to external causes only. Maternal and paternal ages were related to infant death due to both internal and external causes, with the nadir at ages 25–29 years. Among infant characteristics, male, SGA, preterm birth, being a subsequent child, and birth outside of a health care facility were related to deaths due to internal causes as well as external causes. Infant mortality due to both internal and external causes monotonically declined by year of birth, with the exception of a high infant mortality rate due to external causes for those born in 2010 (who would have been under age 1 on the day of the Great East Japan Earthquake).

**Table 2.** Child, parent and household characteristics of infants who survived until their first birthday, infants who died due to internal causes, and infants who died due to external causes among 8,941,501 infants born in Japan in 2003–2010

			Alive		Internal causes			External causes		
			*n* = 8,918,101		*n* = 21,884			*n* = 1,516		
			*n*	%	*n*	%	a)	*n*	%	b)
Child	Sex	Male	4,576,267	51.3%	11,904	54.4%	^***^	874	57.7%	^***^
		Female	4,341,834	48.7%	9,980	45.6%		642	42.3%	
	Multiplicity	Singleton	8,729,002	97.9%	19,827	90.6%	^***^	1,473	97.2%	
		Twin, triplet or higher	189,099	2.1%	2,057	9.4%		43	2.8%	
	SGA	No	8,108,686	90.9%	14,759	67.4%	^***^	1,325	87.4%	^***^
		Yes	658,929	7.4%	6,603	30.2%		142	9.4%	
	Gestational age	37 weeks or above	8,424,180	94.5%	11,410	52.1%	^***^	1,389	91.6%	^***^
		Under 37 weeks	493,921	5.5%	10,474	47.9%		127	8.4%	
	First child	No	4,585,023	51.4%	12,469	57.0%	^***^	868	57.3%	^***^
		Yes	4,333,078	48.6%	9,415	43.0%		648	42.7%	
	Birthplace	Health care facility	8,864,420	99.4%	21,565	98.5%	^***^	1,469	96.9%	^***^
		Home or other	53,681	0.6%	319	1.5%		47	3.1%	
	Year of birth	2003	1,145,834	12.8%	3,259	14.9%	^***^	204	13.5%	^*^
		2004	1,134,372	12.7%	2,982	13.6%		206	13.6%	
		2005	1,086,594	12.2%	2,794	12.8%		205	13.5%	
		2006	1,118,806	12.5%	2,710	12.4%		191	12.6%	
		2007	1,117,353	12.5%	2,769	12.7%		185	12.2%	
		2008	1,119,920	12.6%	2,619	12.0%		166	10.9%	
		2009	1,096,777	12.3%	2,436	11.1%		153	10.1%	
		2010	1,098,445	12.3%	2,315	10.6%		206	13.6%	

Father	Age, years	40 and over	837,797	9.6%	2,883	13.8%	^***^	159	11.2%	^***^
		35–39	1,902,261	21.8%	4,784	23.0%		334	23.5%	
		30–34	3,115,825	35.8%	6,772	32.5%		437	30.8%	
		25–29	2,144,581	24.6%	4,581	22.0%		316	22.3%	
		20–24	667,404	7.7%	1,678	8.1%		162	11.4%	
		19 and under	42,569	0.49%	122	0.59%		11	0.78%	
	Nationality	Japanese	8,498,899	97.6%	20,303	97.5%		1,360	95.8%	^***^
		Non-Japanese	211,544	2.4%	517	2.5%		59	4.2%	
Mother	Age, years	40 and over	210,169	2.4%	1,094	5.0%		45	3.0%	^***^
		35–39	1,477,938	16.6%	4,417	20.2%		239	15.8%	
		30–34	3,312,312	37.1%	7,458	34.1%		479	31.6%	
		25–29	2,748,257	30.8%	5,703	26.1%		420	27.7%	
		20–24	1,036,535	11.6%	2,676	12.2%		266	17.5%	
		19 and under	132,883	1.5%	536	2.4%		67	4.4%	
	Nationality	Japanese	8,683,154	97.4%	21,173	96.8%	^***^	1,420	93.7%	^***^
		Non-Japanese	234,947	2.6%	711	3.2%		96	6.3%	
	Marital status	Married	8,710,437	97.7%	20,820	95.1%	^***^	1,419	93.6%	^***^
		Single, divorced, widowed	207,658	2.3%	1,064	4.9%		97	6.4%	

Household	Employment status	Employed	6,703,467	78.0%	15,478	74.3%	^***^	1,018	70.4%	^***^
		Self-employed, agricultural work or irregular employed	1,683,442	19.6%	4,530	21.7%		346	23.9%	
		Unemployed	209,275	2.4%	836	4.0%		83	5.7%	
	Number of children	1	4,333,078	48.6%	9,415	43.0%	^***^	648	42.7%	^***^
		2–3	4,329,694	48.5%	11,323	51.7%		790	52.1%	
		4 or more	255,329	2.9%	1,146	5.2%		78	5.1%	
	Experienced stillbirth	No	8,870,395	99.5%	21,543	98.4%	^***^	1,502	99.1%	^*^
		Once or more	47,706	0.53%	341	1.6%		14	0.92%	
	Place of residence	Government-designated city	2,236,049	25.1%	5,366	24.5%		352	23.2%	
		Other city	5,524,382	61.9%	13,686	62.5%		949	62.6%	
		Town, village	1,157,670	13.0%	2,832	12.9%		215	14.2%	

^a^Between infants alive at age one and those who died due to internal causes.

^b^Between infants alive at age one and those who died due to external causes.

^*^P < 0.05 ^**^P < 0.01 ^***^P < 0.001. SGA: Small for gestational age. Missing value: SGA (*n* = 151,057: 1.7% of total), paternal age (*n* = 64), maternal age (*n* = 88), single mother (*n* = 6), employment status (*n* = 323,026: 3.6% of total), and number of children (10,419: 0.12% of total).

Table 3 shows the number of deaths by infant age and cause. The total number of deaths significantly decreased with increasing age (*P* for trend <0.001). Half of the subjects (*n* = 11,893, 50.8%) died within the first 4 weeks of life. Among neonatal deaths, over half of deaths due to internal causes and all deaths due to external causes occurred on day 0 or 1; the majority of these (82.7% of deaths by internal causes, and 89.3% of deaths by external causes) occurred on day 0. Deaths due to intentional injuries occurred with the highest frequency on days 0–1 (*n* = 74, 42.3% of neonatal deaths). The number of deaths due to internal causes decreased with increasing weeks of age during the neonatal period, while the number of deaths by unintentional injury and undetermined injury increased.

**Table 3.** Infant deaths by age and causes: analysis of 23,400 infants among those born from 2003–2010

	Total death (*n* = 23,400)^‡^		Death by internal causes (*n* = 21,884)^a^		Death by external causes (*n* = 1,516)^b^		External causes					

							Unintentional injury (*n* = 1,194)		Intentional injury (*n* = 175)		Undetermined injury (*n* = 118)	
Timing of death	*N*	%	*N*	%	*N*	%	*N*	%	*N*	%	*N*	%

Week 0	8,607	36.8%	8,491	38.8%	116	7.7%	30	2.5%	81	46.3%	4	3.4%

Day 0	5,474	23.4%	5,382	24.6%	92	6.1%	19	1.6%	69	39.4%	3	2.5%
Day 1	1,133	4.8%	1,122	5.1%	11	0.73%	5	0.42%	5	2.9%	1	0.85%
Day 2–6	2,000	8.5%	1,987	9.1%	13	0.86%	6	0.50%	7	4.0%	0	0.00%

Week 1–3	3,286	14.0%	3,217	14.7%	69	4.6%	48	4.0%	10	5.7%	5	4.2%
Week 4–7	2,171	9.3%	2,044	9.3%	127	8.4%	103	8.6%	15	8.6%	9	7.6%
Week 8–11	1,525	6.5%	1,370	6.3%	155	10.2%	135	11.3%	9	5.1%	9	7.6%
Week 12–15	1,254	5.4%	1,114	5.1%	140	9.2%	120	10.1%	8	4.6%	11	9.3%
Week 16–19	1,153	4.9%	1,021	4.7%	132	8.7%	110	9.2%	8	4.6%	14	11.9%
Week 20–23	1,047	4.5%	897	4.1%	150	9.9%	126	10.6%	6	3.4%	17	14.4%
Week 24–27	913	3.9%	774	3.5%	139	9.2%	108	9.0%	15	8.6%	13	11.0%
Week 28–31	799	3.4%	682	3.1%	117	7.7%	102	8.5%	4	2.3%	10	8.5%
Week 32–35	683	2.9%	596	2.7%	87	5.7%	73	6.1%	7	4.0%	6	5.1%
Week 36–39	561	2.4%	484	2.2%	77	5.1%	67	5.6%	4	2.3%	5	4.2%
Week 40–43	507	2.2%	436	2.0%	71	4.7%	60	5.0%	4	2.3%	4	3.4%
Week 44–47	445	1.9%	379	1.7%	66	4.4%	56	4.7%	3	1.7%	4	3.4%
Week 48–52	447	1.9%	377	1.7%	70	4.6%	56	4.7%	1	0.6%	7	5.9%

^‡^Nonparametric trend test performed for total deaths among every 4 weeks of infant age. *P* for trend <0.001.

^a^Two infants were not included due to missing of timing of death by internal causes.

^b^29 infants were not included died from non-injury related external causes (ie, medical treatment related deaths, such as medications or surgeries).

When background characteristics for deaths during the neonatal period were compared by timing of death, we found that births outside of health care facilities had a higher proportion of early neonatal deaths due to both internal and external causes, especially for deaths at days 0–1, compared to births in the later neonatal period (days 2–6 and days 7–27). The proportion of preterm infants among deaths due to internal causes was higher in the early neonatal period (days 0–1 and days 2–6) than the late neonatal period (days 7–27), while the proportion of SGA infants was highest for deaths at days 2–6. The proportion of infants born to single mothers among deaths due to internal causes was higher at days 0–1 compared to later in the neonatal period, while the proportion of infants from employed households was higher among deaths later in the neonatal period. For deaths due to external causes, multiplicity, maternal nationality, maternal marital status, and number of children were significantly different between deaths at different periods (eTable 1). When deaths on days 0 and 1 were compared, deaths due to internal causes on day 0 were more likely to be related to preterm, SGA, and birth outside a health care facility than those on day 1. However, we failed to detect any significant difference in the background characteristics of patients who died on days 0 and 1 in terms of external causes and intentional injury (data not shown).

**Figure 1.**
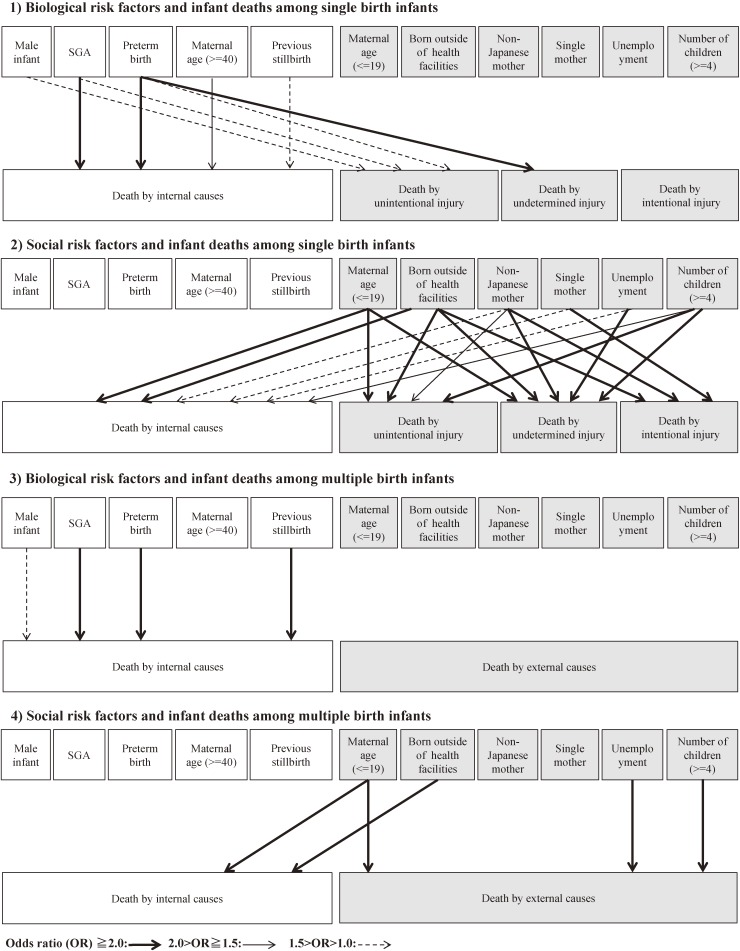
Association between biological or social risk factors and infant deaths by causes. Analysis of 8,941,501 infants born in Japan in 2003–2010.

Figure 1 illustrates relationships between biological or social risk factors and cause-specific infant deaths from the multivariable logistic regression model, with eTable 2 showing the estimated adjusted odds ratios (ORs) as well as the 95% confidence intervals (CIs).

For singleton infants, the biological risk factors associated with at least a two times higher risk of death were SGA (OR 4.4; 95% CI, 4.3–4.6) and preterm birth (OR 14.5; 95% CI, 14.1–15.0) for internal causes, and preterm birth (OR 2.7; 95% CI, 1.5–4.8) for undetermined injury.

All social risk factors—young maternal age (≤19 years), birth outside of a health care facility, having a non-Japanese mother or single mother, living in a unemployed household, and having four or more children in the household—were significant risk factors for death by internal causes. Among these factors, birth outside of a health care facility (OR 3.2; 95% CI, 2.7–3.8) was the only factor associated with at least a two times higher risk.

Social risk factors showing an increased risk of unintentional injury by at least two-fold were maternal age of 20–24 years (OR 2.0; 95% CI, 1.7–2.4), maternal age of 19 years and under (OR 4.1; 95% CI, 3.0–5.7), and having a high number of children in the household (OR 2.5; 95% CI, 1.8–3.3). Similarly, for undetermined injury, the factors were birth outside of a health care facility (OR 6.5; 95% CI, 1.6–26.5), maternal age of 19 years and under (OR 3.7; 95% CI, 1.4–9.8), having a non-Japanese mother (OR 2.8; 95% CI, 1.3–5.8), living in an unemployed household (OR 3.3; 95% CI, 1.5–7.3), and having a high number of children in the household (OR 3.4; 95% CI, 1.6–7.3). For intentional injury, the factors were birth outside of a health care facility (OR 15.9; 95% CI, 7.0–36.3), having a non-Japanese mother (OR 6.9; 95% CI, 4.4–10.8) and single mother (OR 3.0; 95% CI, 1.5–5.9).

For multiple births, the biological risk factors associated with at least a two times higher risk of death were SGA (OR 2.1; 95% CI, 2.0–2.4), preterm birth (OR 5.6; 95% CI, 4.9–6.4), and experience of stillbirth (OR 5.1; 95% CI, 4.0–6.5) for internal causes. Social risk factors showing an increased risk of death due to internal causes at least two-fold were birth outside of a health care facility (OR 14.3; 95% CI, 5.8–35.6) and maternal age of 19 years and under (OR 2.1; 95% CI, 1.4–3.0). For deaths due to external causes, social risk factors showing at least a two times higher risk were maternal age of 19 years and under (OR 11.7; 95% CI, 2.4–55.8), living in an unemployed household (OR 4.5; 95% CI, 1.2–17.4), and having a high number of children in the household (OR 3.0; 95% CI, 1.1–8.5). On the other hand, none of the biological risk factors of interest doubled risk of death by external causes.

**Figure 2.**
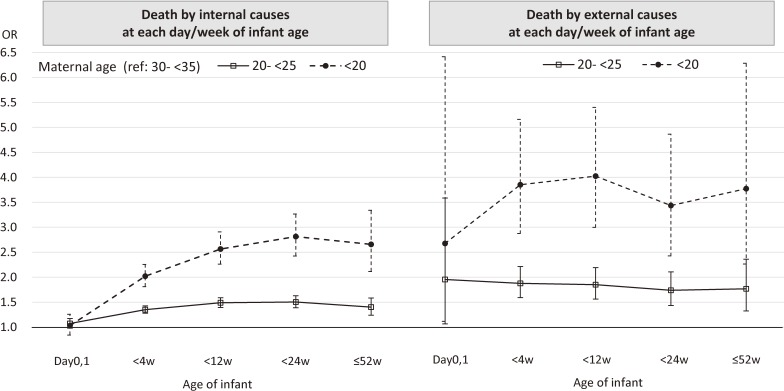
Young maternal age and infant death at each day/week of infant age compared to mothers aged 30–34 years old.

In Figure 2, we show the association between young maternal age and infant mortality at various time intervals (fulll multi-nominal logistic regression models by each time intervals were not shown in eTable 2). Infants with mothers aged 20 to 25 years old, or 19 years and under, had a significantly higher risk of death due to internal causes as well as external causes at all time periods, except for death due to internal causes at days 0–1. However, while the effect of younger maternal age was steadily around three to four times higher for death due to external causes, the adverse effect of younger maternal age on death due to internal causes gradually rose as infant age increased up until 6 months of age.

## DISCUSSION

This is the first study to comprehensively and simultaneously examine the effect of biological and social risk factors on cause-specific infant deaths. Infants with high social risk (births outside of a health care facility; or to teenage, non-Japanese, or single mothers; or from an unemployed household or household with four or more children) had a higher risk of death not only due to external causes, but also due to internal causes. Infants with biological risk factors (SGA and preterm infants) had a higher risk of death due to disease as well as external causes. Young mothers have increased risk of death due to disease, especially during the later period of infanthood.

### Biological risk factors surrounding infant death

In our study, we found that for both singletons and multiples, infants who were born SGA, preterm, or to women with prior experience of stillbirth showed an increased risk of death due to internal causes. Advanced age of the mother (40 years and older) was a significant risk factor only for singletons, while male infants were significantly related to deaths by internal causes among multiple births. These risk factors are consistent with previous studies examining infant all-cause mortality.^5^^,^^26^^–^^28^

Biological risk factors were related not only to deaths due to internal causes, but also to those due to external causes. We found that male, SGA, and preterm infants were at higher risk of death due to unintentional injury, and preterm infants were at higher risk of death due to undetermined injury among singleton infants. We failed to find significant associations between biological risk factors and unintentional or intentional injury of death among multiple birth infants; however, this may have been due to the small number of external causes of death in our study. Two previous studies on linked birth and death data in the United States^19^^,^^29^ reported that being male and of low birth weight (LBW) were risk factors for fatal unintentional injury, which is consistent with our findings. Such characteristics of premature infants are known to increase the risk of postpartum depression,^10^ adversely influencing parent-infant interaction, child safety practice,^30^ and quality of maternal supervision.^31^ Thus, our findings suggest that for such infants, health professionals should pay attention not only to the child’s health condition, but also to provide the family supportive resources to prevent dangerous situations that could lead to unintentional or intentional injuries. In our study, we failed to find prematurity of infants to be a risk factor of intentional injury, which was shown in a United States study.^19^^,^^29^ This disparity in findings may have been due to the fact that the previous study did not concurrently adjust for birth outside of a health care facility, a possible confounder strongly associated with both LBW/preterm delivery and intentional injury. In our study, nearly 40% of intentional injuries occurred on day 0, suggesting unexpected birth is a strong driving factor.

### Social risk factors surrounding infant death

In our study, children born to teenage mothers, in unemployed households, and in households with a high number of children had significantly higher risk of death by external causes for both singletons and multiples. We also found that having a non-Japanese mother and giving birth outside of a health care facility were significantly related to external causes of death only among singleton infants. For deaths by internal causes, teenage pregnancy and birth outside of a health care facility were significant risk factors for internal causes of deaths among infants of both singleton and multiple births. Having a single or non-Japanese mother and being born in an unemployed household or a household with a high number of children were significantly associated with death due to internal causes only among singleton infants.

Although no previous study has specifically looked at the association between social factors and infant death due to internal causes as in our study, our findings are similar to those from studies reporting that infants of teenaged and unmarried mothers,^11^^–^^13^^,^^15^ and of a higher order of birth,^13^^–^^15^ had increased risk of death due to specific internal causes, including lower respiratory tract infection,^12^ diarrhea,^13^ intussusception,^14^ and necrotising enterocolitis.^15^ Socially high-risk mothers tend to be isolated and have less resources to obtain knowledge on child-caring or ask for support when necessary, which may inhibit them from seeking medical care when needed.

Among social risk factors, birth outside of a health care facility was most strongly associated with death by intentional injury. Previous research has also shown that delivery outside of a health care facility increases risk of neonaticide.^32^^,^^33^ However, interestingly, we found that this group of children retains a higher risk for unintended injury and death by internal causes, even beyond the neonatal period. Women who deliver outside of a health care facility share backgrounds with mothers who did not receive prenatal care^34^ due to out-of-pocket expenses, had a lack of knowledge about prenatal care, or had an unwanted pregnancy, including those women who wanted to have an abortion but were not able to.^35^ To prevent infant deaths, our study suggests that health professionals need to provide continuous support on childrearing to mothers who delivered outside of a health care facility, even beyond the neonatal period.

### Young maternal age

Young maternal age has been reported to be a significant risk factors for falls,^36^ traffic accidents,^36^ neonaticide,^33^^,^^34^ and child abuse and neglect.^37^^,^^38^ In our study, young maternal age, especially teenage mothers and those in their early twenties, showed a significantly higher risk of both external and internal causes of infant death. In addition, we found that risk of infant mortality by internal causes due to younger maternal age increased with infant age. This phenomenon may reflect that parenting difficulties or a lack of care-seeking behavior among young mothers becomes more apparent in the later months of infancy. During this period, childcare becomes more eventful, as infants start to move around, begin eating solids, and become more prone to developing fevers, and younger mothers may not be able to keep up with the increased demand in parenting skills. Understanding the difficulties young mothers are facing and providing opportunities to receive adequate support and information may not only be important in the beginning of infancy, but also in the later infantile period.

### Limitations and future directions

A key strength of this study is its focus on both social and biological factors simultaneously to provide a comprehensive assessment of how such risk factors relate to cause-specific deaths among infants, using a nationwide survey of all births in Japan. Our findings emphasise the importance of paying attention to risk factors of infant death by both internal and external causes for those infants living in socio-demographic and socio-economic risk factors, as well as for infants with biological risk factors that increase the risk of severe disease.

However, our study has several limitations. First, as we used ICD-10 codes from the death certificates (filled in by the physician who confirmed the death) to classify cause of death, miscoding may have occurred; for example, death from abuse or neglect may be overlooked and mistakenly diagnosed as death from internal causes. Second, although we used a linkage process that successfully linked over 99.9% of the death certificates to birth certificates, our linkage process relied on a combination of common variables. Although previous reports have shown this method to be possibly more valid than deterministic record linkage,^39^ we were not able to link records using unique identifiers as has been done in other countries, such as the United States. Third, as we derived the timing of death by subtracting time of birth as reported on the birth certificate from time of death reported on the death certificate, the accuracy of the timing may have been affected by misreporting of timing of birth, especially of unattended births occurring outside a hospital. Such misclassification would likely influence the calculation of the timing of death on day 0–1, and may explain why we failed to find any difference in background characteristics between infants who died on day 0 or day 1 due to intentional injury or external causes of death. Fourth, as our analysis was limited to social variables derived from the birth certificate, we could not evaluate other important socio-economic factors, such as income, education, residence, or neighborhood situations, or other more personal factors related to child-rearing, such as maternal mental health, co-residence with other family members, relationships between family members, and perceptions toward childrearing. To evaluate such detailed information, a multi-disciplinary system collecting information from a wide range of resources, such as the Child Death Review system, is needed.^4^^,^^40^ If preventive interventions are implemented in the future, follow-up studies are also needed to evaluate changes in risk factors for infant deaths.

### Conclusion

Infants with biological risk factors had a higher risk of death from unintentional external causes as well as internal causes, and infants of socially high-risk mothers had a high risk of death from both external and internal causes. Interdisciplinary support from both public health and clinical-care professionals is needed to prevent diseases and injuries among infants with high social or biological risks.

## APPENDIX A. SUPPLEMENTARY DATA

Appendix 1. Data linkage method between birth and infant death certificates

eTable 1. Differences between infant death of early neonate (day 0–1, day 2–6) and late neonate (day 7–27)

eTable 2. Factors related to causes specific deaths stratified by single or multiple births compared to living infants
