# Supplementary material for: Comprehensive Assessment of Risk Factors of Cause-Specific Infant Deaths in Japan
Source: J Epidemiol. 2018 Jun 5;28(6):307–14. doi: 10.2188/jea.JE20160188 (PMC5976875; doi:10.2188/jea.JE20160188)
Supplement: Supplementary file 1 [file je-28-307-s001.pdf]

Appendix 1. Data linkage method between birth and infant death certificates

The birth and infant death certificates of Japan have the following variables in common: infant nationality (Japanese, non-Japanese), day of birth, day of mother's birth, sex, gestational age (in weeks), birth weight, multiplicity, birth order and sex. However, while birth certificates are filled in by physicians attending birth, infant death records are filled in by medical examiners or physicians stating death, thus in death certificates data on birth characteristics (gestational age, birth weight, multiplicity and birth order) as well as mother's day of birth, are more likely to be erroneous or missing compared to those reported in birth certificates. In our dataset we were able to match only 88% of the death certificates to a matching birth certificate by using deterministic data linkage.

Therefore, we used the probabilistic linkage method developed by Fellegi and Sunter[16] to link the two sets of data. This method takes into account data quality and data uniqueness to estimate the "match probability" that each case in a dataset matches each case in a different database, and determines whether the two cases "match" by comparing the "match probability" to a threshold set *a priori*. The "match probability" is calculated from two probabilities: the probability that two records belonging to different people coincidentally agree with each other, which is calculated from the distribution of the records data (u-probability), and the probability that two records belonging to the same person disagree with each other due to data entry errors, which is decided from quality of the data of interest (m-probability).

We set the m-probability for each variable as below: day of birth 0.99, sex 0.99, gestational age (in weeks) 0.98, birth weight 0.95, multiplicity 0.95, birth order 0.95, day of mother's birth 0.90 and infant nationality 0.90. As we assumed data entry mistakes would be more likely to switch numbers (such as enter birth weight as 925 g instead of 952 g, or enter birth date as 9/3/2012 instead of 3/9/2012) rather than enter unrelated numbers, each of these variables were treated as strings of characters, and Levenshtein distance was used to measure the difference between two sequences. The match probability threshold was set to 0.5 to maximize the probability each death certificate would match at least one birth certificate. As we assumed all deaths to have one corresponding birth certificate, we conducted a one-to-multiple-record match, which would choose the death certificate with highest match probability for each birth certificate. Using this method all birth certificates would identify no more than one death certificate each as its match, but multiple birth certificates would be allowed to identify the same death certificate as its match. All analyses were conducted using LinkPlus Beta version (U.S. Centers for Disease Control and Prevention, Cancer

Division, Washington, DC, USA).

Among 100,175,196 birth certificates, 25,413 were uniquely matched with one death certificate each, and 22 birth certificates were matched to 11 death certificates (two each). Thirty eight death certificates were not matched to any birth certificate. Thus, we excluded the 22 (<0.001% among 100,175,196) birth certificates which were matched to multiple death certificates and 49 (0.2% among 25,491) death certificates which were not uniquely matched to a birth certificate, and used the remaining 100,175,174 birth certificates linked to 25,413 infant death certificates for the analysis.

eTable 1. Differences between infant death of early neonate (day 0–1, day 2–6) and late neonate (day 7–27)

	Death by internal cause (n=11,708)							Death by external cause (n=185)							
	Day 0–1 (n=6,504)		Day2–6 (n=1,987)		Day7–27 (n=3,217)			Day 0–1 (n=103)		Day 2–6 (n=13)		Day 7–27 (n=69)			
	n	%	n	%	n	%	p-value	n	%	n	%	n	%	p-value	
Male infant	3,509	54.0%	1,134	57.1%	1,731	53.8%	**0.035**	46	44.7%	7	53.8%	40	58.0%	0.604	†
Multiplicity	747	11.5%	280	14.1%	418	13.0%	**0.004**	0	0.0%	0	0.0%	4	5.8%	0.058	†
Birth outside of health care facility	179	2.8%	21	1.1%	25	0.8%	**<0.001**	38	36.9%	1	7.7%	0	0.0%	**<0.001**	†
Preterm	4,171	64.1%	1,263	63.6%	1,877	58.3%	**<0.001**	4	3.9%	2	15.4%	5	7.2%	0.160	†
SGA	1,922	29.6%	683	34.4%	961	29.9%	**<0.001**	7	6.8%	0	0.0%	8	11.6%	0.650	†
Maternal age, years ≥40	313	4.8%	103	5.2%	172	5.3%		8	7.8%	1	7.7%	1	1.4%		†
35–39	1,365	21.0%	433	21.8%	660	20.5%		17	16.5%	2	15.4%	14	20.3%		
30–34	2,238	34.4%	712	35.8%	1,115	34.7%	**0.028**	27	26.2%	3	23.1%	18	26.1%	0.227	
25–29	1,765	27.1%	491	24.7%	842	26.2%		22	21.4%	3	23.1%	22	31.9%		
20–24	703	10.8%	196	9.9%	381	11.8%		21	20.4%	2	15.4%	14	20.3%		
≤19	120	1.8%	52	2.6%	47	1.5%		8	7.8%	0	0.0%	0	0.0%		
Non-Japanese mother	205	3.2%	69	3.5%	94	2.9%	0.541	23	22.3%	0	0.0%	1	1.4%	**<0.001**	†
Single mother	390	6.0%	108	5.4%	135	4.2%	**0.001**	35	34.0%	1	7.7%	1	1.4%	**<0.001**	†
Experience of stillbirth	128	2.0%	38	1.9%	62	1.9%	0.983	0	0.0%	0	0.0%	1	1.4%	0.443	†
Working status of household:															
Employment	4,482	68.9%	1,409	74.4%	2,339	76.4%	**0.002**	55	53.4%	8	61.5%	46	66.7%	0.095	†

Self-employed	1,467	22.6%	422	22.3%	621	20.3%		24	23.3%	3	23.1%	16	23.2%	
No job	232	3.6%	62	3.3%	103	3.4%		18	17.5%	1	7.7%	3	4.3%	
Number of children														
1	2,983	45.9%	917	46.1%	1,448	45.0%		63	61.2%	6	46.2%	26	37.7%	†
2–3	3,192	49.1%	964	48.5%	1,607	50.0%	0.849	35	34.0%	7	53.8%	37	53.6%	**0.032**
≥ 4	303	4.7%	99	5.0%	152	4.7%		5	4.9%	0	0.0%	6	8.7%	
Place of Residence														
Designated city	1,598	24.6%	450	22.6%	836	26.0%		24	23.3%	3	23.1%	14	20.3%	†
City	4,042	62.1%	1,246	62.7%	1,969	61.2%	0.054	53	51.5%	5	38.5%	46	66.7%	0.103
Town or village	864	13.3%	291	14.6%	412	12.8%		26	25.2%	5	38.5%	9	13.0%	
Year														
2003	1,015	15.6%	303	15.2%	564	17.5%		17	16.5%	1	7.7%	8	11.6%	†
2004	887	13.6%	310	15.6%	447	13.9%		12	11.7%	3	23.1%	9	13.0%	
2005	834	12.8%	274	13.8%	410	12.7%		17	16.5%	1	7.7%	13	18.8%	
2006	784	12.1%	281	14.1%	386	12.0%	**<0.001**	13	12.6%	2	15.4%	8	11.6%	0.834
2007	826	12.7%	243	12.2%	378	11.8%		8	7.8%	3	23.1%	9	13.0%	
2008	759	11.7%	201	10.1%	364	11.3%		19	18.4%	2	15.4%	8	11.6%	
2009	709	10.9%	181	9.1%	377	11.7%		10	9.7%	0	0.0%	8	11.6%	
2010	690	10.6%	194	9.8%	291	9.0%		7	6.8%	1	7.7%	6	8.7%	

SGA, small for gestational age.
† Fisher exact test used instead of chi-square test.

eTable 2. Factors related to causes specific deaths stratified by single or multiple births compared to living infants

2-1. Single birth infants (n=8,423,912)

	All death				Death by internal cause				Death by external cause				Unintentional injury death				Undetermined injury death				Intentional injury death			
	OR	95% CI			OR	95% CI			OR	95% CI			OR	95% CI			OR	95% CI			OR	95% CI		
Infant characteristics																								
Male	**1.04**	**1.02**	-	**1.07**	1.03	0.99	-	1.06	**1.27**	**1.14**	-	**1.42**	**1.35**	**1.19**	-	**1.52**	1.12	0.77	-	1.64	1.03	0.74	-	1.43
Birth outside of health care facility	**3.26**	**2.79**	-	**3.80**	**3.20**	**2.72**	-	**3.76**	**4.23**	**2.54**	-	**7.06**	**2.52**	**1.19**	-	**5.30**	**6.48**	**1.58**	-	**26.5**	**15.9**	**6.95**	-	**36.3**
SGA	**4.18**	**4.05**	-	**4.31**	**4.42**	**4.28**	-	**4.56**	**1.25**	**1.03**	-	**1.51**	**1.26**	**1.02**	-	**1.56**	1.46	0.78	-	2.72	0.93	0.47	-	1.83
Preterm birth	**13.1**	**12.8**	-	**13.5**	**14.5**	**14.1**	-	**15.0**	**1.49**	**1.21**	-	**1.84**	**1.34**	**1.05**	-	**1.71**	**2.67**	**1.49**	-	**4.79**	1.46	0.77	-	2.80
Maternal characteristics																								
Maternal age, years (ref: 30-34)																								
≥40	**1.64**	**1.53**	-	**1.76**	**1.66**	**1.55**	-	**1.79**	1.09	0.77	-	1.55	0.98	0.65	-	1.49	1.24	0.43	-	3.52	1.78	0.70	-	4.54
35–39	**1.15**	**1.11**	-	**1.20**	**1.16**	**1.11**	-	**1.21**	1.04	0.88	-	1.23	1.02	0.85	-	1.23	0.78	0.43	-	1.42	1.57	0.96	-	2.54
25–29	1.00	0.97	-	1.04	1.00	0.96	-	1.04	1.11	0.97	-	1.28	**1.17**	**1.00**	-	**1.36**	0.86	0.52	-	1.42	1.05	0.68	-	1.64
20–24	**1.31**	**1.25**	-	**1.37**	**1.27**	**1.21**	-	**1.33**	**1.88**	**1.59**	-	**2.21**	**2.02**	**1.69**	-	**2.42**	1.69	0.96	-	2.98	1.36	0.79	-	2.32
≤19	**1.85**	**1.69**	-	**2.04**	**1.74**	**1.57**	-	**1.93**	**3.66**	**2.74**	-	**4.89**	**4.14**	**3.00**	-	**5.71**	**3.67**	**1.38**	-	**9.76**	1.60	0.60	-	4.28
Non-Japanese mother	**1.27**	**1.17**	-	**1.38**	**1.19**	**1.09**	-	**1.30**	**2.31**	**1.83**	-	**2.92**	**1.63**	**1.20**	-	**2.22**	**2.79**	**1.33**	-	**5.81**	**6.86**	**4.35**	-	**10.8**
Single mother	**1.28**	**1.18**	-	**1.39**	**1.30**	**1.19**	-	**1.42**	1.14	0.84	-	1.55	1.03	0.70	-	1.49	0.51	0.16	-	1.61	**3.00**	**1.54**	-	**5.85**
Experience of stillbirth	**1.25**	**1.08**	-	**1.45**	**1.24**	**1.07**	-	**1.44**	1.43	0.74	-	2.77	1.22	0.55	-	2.73	1.71	0.24	-	12.3				
Household characteristics																								
Working status (ref: employment)																								
Self employed	**1.11**	**1.07**	-	**1.15**	**1.10**	**1.06**	-	**1.14**	**1.26**	**1.11**	-	**1.43**	**1.29**	**1.12**	-	**1.48**	1.48	0.95	-	2.30	1.03	0.67	-	1.57
Unemployment	**1.19**	**1.10**	-	**1.30**	**1.16**	**1.07**	-	**1.27**	**1.59**	**1.19**	-	**2.11**	1.32	0.93	-	1.87	**3.30**	**1.49**	-	**7.31**	1.83	0.92	-	3.66
Number of children (ref: 1)																								
2–3	**1.23**	**1.20**	-	**1.27**	**1.22**	**1.19**	-	**1.26**	**1.42**	**1.27**	-	**1.60**	**1.53**	**1.34**	-	**1.74**	1.31	0.86	-	1.98	0.93	0.66	-	1.33
≥4	**1.53**	**1.42**	-	**1.64**	**1.50**	**1.39**	-	**1.61**	**2.18**	**1.68**	-	**2.84**	**2.45**	**1.82**	-	**3.29**	**3.40**	**1.58**	-	**7.34**	0.62	0.22	-	1.76

CI. confidence interval; OR, odds ratio.
All models were adjusted by place of residence and year.

2-2. Multiple birth infants (n=184,527)

	All death				Death by internal cause				Death by external cause			
	OR	95% CI			OR	95% CI			OR	95% CI		
Infant characteristics												
Male	**1.19**	**1.09**	-	**1.30**	**1.19**	**1.09**	-	**1.31**	0.97	0.52	-	1.81
Birth outside of health care facility	**14.3**	**5.76**	-	**35.3**	**14.3**	**5.76**	-	**35.6**	-	-		-
SGA	**2.13**	**1.94**	-	**2.34**	**2.14**	**1.95**	-	**2.35**	1.77	0.93	-	3.36
Preterm birth	**5.37**	**4.71**	-	**6.11**	**5.63**	**4.93**	-	**6.44**	1.22	0.64	-	2.30
Maternal characteristics												
Maternal age, years (ref: 30-34)												
≥40	0.95	0.72	-	1.25	0.95	0.72	-	1.25	1.07	0.14	-	8.33
35–39	1.07	0.95	-	1.21	1.06	0.94	-	1.20	1.73	0.75	-	4.00
25–29	**1.20**	**1.07**	-	**1.35**	**1.19**	**1.06**	-	**1.33**	1.95	0.87	-	4.36
20–24	**1.62**	**1.38**	-	**1.89**	**1.62**	**1.38**	-	**1.90**	1.06	0.23	-	4.83
**≤19**	**2.18**	**1.49**	-	**3.19**	**2.05**	**1.39**	-	**3.04**	**11.7**	**2.43**	-	**55.8**
Non-Japanese mother	1.00	0.73	-	1.37	1.02	0.75	-	1.40	-	-		-
Single mother	1.22	0.87	-	1.73	1.26	0.89	-	1.79	0.39	0.04	-	3.74
Experience of stillbirth	**5.01**	**3.94**	-	**6.36**	**5.12**	**4.03**	-	**6.51**	-	-		-
Household characteristics												
Working status (ref: employment)												
Self employed	**1.16**	**1.04**	-	**1.30**	**1.17**	**1.05**	-	**1.31**	0.79	0.33	-	1.89
Unemployment	0.99	0.71	-	1.38	0.94	0.66	-	1.32	**4.53**	**1.18**	-	**17.4**
Number of children (ref: 1)												
2–3	0.91	0.82	-	1.00	0.91	0.82	-	1.00	1.06	0.50	-	2.23
≥4	1.16	0.96	-	1.40	1.13	0.93	-	1.37	**2.99**	**1.06**	-	**8.47**

CI, confidence interval; OR, odds ratio.
All models were adjusted by place of residence and year.
